# Vascular endothelial growth factor A is a potential prognostic biomarker and correlates with immune cell infiltration in hepatocellular carcinoma

**DOI:** 10.1111/jcmm.17678

**Published:** 2023-02-02

**Authors:** Yuchen Qi, Yinghui Song, Mengting Cai, Jianwen Li, Zhangtao Yu, Yuhang Li, Junkai Huang, Yu Jiang, Chuang Peng, Bo Jiang, Sulai Liu

**Affiliations:** ^1^ Department of Hepatobiliary Surgery Hunan Provincial People's Hospital/The First Affiliated Hospital of Hunan Normal University Changsha China; ^2^ Department of Cardiology Xiangdong Hospital Affiliated to Hunan Normal University Liling China; ^3^ Central Laboratory of Hunan Provincial People's Hospital/The First Affiliated Hospital of Hunan Normal University Changsha China; ^4^ Department of Nuclear Medicine Hunan Provincial People's Hospital/The First Affiliated Hospital of Hunan Normal University Changsha China; ^5^ Institute of Emergency Medicine/Hunan Provincial Key Laboratory of Emergency and Critical Care Metabonomics Hunan Provincial People's Hospital/The First Affiliated Hospital of Hunan Normal University Changsha China

**Keywords:** hepatocellular carcinoma, immune infiltrates, prognostic biomarker, VEGFA

## Abstract

Hepatocellular carcinoma (HCC) is the fourth leading cause of cancer‐related deaths among cancer patients. Vascular endothelial growth factor A (VEGFA) is involved in regulating biological processes, such as angiogenesis and vascular permeability, and is very closely related to the pathogenesis of various tumours, especially vascular‐rich, solid tumours. Clinical data of patients with HCC and other tumours were analysed through public databases, such as the TCGA database, Gene Expression Omnibus database, Human Protein Atlas database, STRING, Tumour Immune Estimation Resource and Kaplan–Meier Plotter. The tumour tissues and adjacent normal tissues of patients with HCC from Hunan Provincial People's Hospital were collected to verify the expression of VEGFA by immunohistochemistry, immunofluorescence, Western blotting and qPCR. VEGFA expression is elevated in multiple tumour types and correlates with the prognosis of tumour patients. VEGFA is involved in regulating the tumour microenvironment and immune cell function in tumour development. Inhibition of VEGFA reduces proliferation, invasion, and migration and promotes apoptosis in HCC cells. VEGFA is a potential predictive biomarker for the diagnosis and prognosis of HCC.

## 1 BACKGROUND

Hepatocellular carcinoma (HCC) is one of the most common malignancy and is currently one of the leading causes of cancer‐related death.^1^, ^2^, ^3^ More than 700,000 people die of HCC every year worldwide.^4^ China has the greatest number of cases of HCC in the world.^3^, ^5^ The most important risk factors for HCC include chronic infection with hepatitis B virus or hepatitis C virus and exposure to aflatoxin.^5^, ^6^, ^7^ Surgical resection is the most effective treatment for patients with HCC,^8^, ^9^ but the risk of recurrence 5 years after surgical resection is as high as 70%; further, relapse within 2 years is more likely.^10^, ^11^ Moreover, most patients with HCC miss the opportunity for radical surgery because they are usually diagnosed as intermediate and advanced stages.^11^, ^12^ Comprehensive treatment, including radiotherapy, interventional therapy, targeted therapy and immunotherapy, is very important for postoperative recurrence and inoperable HCC. In recent years, an increasing number of clinical studies have explored the efficacy of immunotherapy for HCC.^13^, ^14^ However, our understanding of immunotherapy for HCC is still insufficient. Therefore, it is of great significance to find biomarkers related to the prognosis and immune infiltration of HCC.

It is believed that tumour growth is controlled by tumour angiogenesis.^15^ Angiogenesis is one of the malignant features of tumours.^16^ The switch of tumour angiogenesis is induced by angiogenic factors secreted by tumour cells or stromal cells, and VEGF is the strongest angiogenesis stimulator.^17^, ^18^ There are five kinds of VEGF, namely VEGFA, VEGFB, VEGFC, VEGFD and PIGF.^19^, ^20^ The combination of Vascular endothelial growth factor A (VEGFA) and VEGFR2 is mainly involved in the regulation of angiogenesis.^21^ VEGFA combined with VEGFR2 triggers signalling cascade pathways and ultimately induces endothelial cell proliferation, survival and migration to promote tumour progression.^22^, ^23^ Sorafenib, a commonly used targeted drug in HCC, also inhibits angiogenesis as an important mechanism.^24^, ^25^ Recent studies have illustrated that VEGFA is highly expressed in malignant tumours, including HCC.^26^, ^27^ The expression and predictive significance of VEGFA in HCC need to be further studied.

In this study, the expression of VEGFA and its relationship with prognosis in multiple malignant tumours were analysed by the The Cancer Genome Atlas Program (TCGA), Kaplan–Meier plotter, Gene Expression Profiling Interactive Analysis (GEPIA), The Human Protein Atlas (HPA), Genotype‐Tissue Expression (GTEx), Gene Expression Omnibus (GEO), European Genome‐phenome Archive (EGA) and Tumour Immune Estimation Resource (TIMER) databases. Next, the relationship between VEGFA and tumour‐infiltrating immune cells (TIICs) was also revealed. Moreover, VEGFA expression was verified by PCR, Western blotting and immunohistochemistry (IHC) in HCC. The function of VEGFA was explored in HCC cell lines. The results demonstrated that VEGFA could play an important role in the prognosis of HCC. This finding also suggested that VEGFA might regulate the infiltration of immune cells in HCC.

## 2 MATERIALS AND METHODS

### 2.1 Data resources for clinical and pathological information

The data are from public databases, including TCGA website (https://genome‐cancer.ucsc.edu/) and The HPA (http://www.proteinatlas.org/). TCGA is a landmark cancer genomics program that provides molecular characterization of tumour samples and matched normal samples of more than 20,000 primary cancers across 33 cancer types. Clinical information on patients with HCC and high‐throughput RNA‐sequencing data were downloaded from the TCGA database. The transcript expression levels were estimated using the fragments per kilobase per million fragments mapped (FPKM) method in HTSeq. HPA is a Swedish initiative launched in 2003 to map all human proteins in cells, tissues and organs using the integration of various omics technologies, including antibody‐based imaging, mass spectrometry‐based proteomics and transcriptome science; the initiative also provides free access to immunohistochemical images of human‐related tumour tissues and corresponding normal tissues. In addition, HCC tissue samples and adjacent non‐tumour tissue samples were obtained from patients diagnosed with HCC at the Department of Hepatobiliary Surgery, Hunan Provincial People's Hospital/The First Affiliated Hospital of Hunan Normal University as described in a previous study.^28^ All cancer tissue samples were pathologically confirmed as HCC by two pathologists.

### 2.2 Analysis of survival data and drawing of ROC curve

The Gepia2 website (http://gepia2.cancer‐pku.cn/) was applied to analyse the survival data related to different cancer patients in the GTEx database (www.gtexportal.org). The influence of the VEGFA gene expression level on the prognosis of each tumour was analysed. Then, the survival data on patients with HCC were obtained from the TCGA database. According to the median VEGFA mRNA expression, all patients with HCC were divided into a VEGFA mRNA high expression group and a VEGFA mRNA low expression group. Finally, the Kaplan–Meier survival curve was drawn by the survminer package and the survival package to analyse the effect of the expression level of the VEGFA gene on the clinical prognosis of patients with HCC. Furthermore, the results from the TCGA database were verified again through the Kaplan–Meier Plotter website. The Kaplan–Meier Plotter website (www.kmplot.com/) is able to assess the impact of 54 k genes (mRNA, miRNA, protein) on the survival of 21 cancer types, including HCC. The website data come from GEO, EGA and TCGA. Then, the clinical, diagnostic effects of VEGFA and AFP were compared by the pROC package and the ggplot2 package, and the ROC curve was drawn.

### 2.3 Univariate and multivariate logistic regression analysis

The variables including age, T stage, N stage, M stage, pathologic stage, histologic grade, adjacent hepatic tissue inflammation, vascular invasion, sex and VEGFA were input to further analyse the influence of clinicopathological features on the prognosis of HCC. The hazard rate (95% CI) was analysed by the survival package using univariate and multivariate analyses, and *p* values were calculated. Finally, prognostic predictors on patients with HCC were obtained.

### 2.4 GSEA and GO KEGG analysis

In this study, we analysed the correlation between VEGFA mRNA expression and all other genes. The clusterProfiler package was applied for GSEA, and the org.Hs.e.g.db package and clusterProfiler package were used for gene ontology (GO) Kyoto Encyclopedia of Genes and Genomes (KEGG) analysis. |ES| > 1, *p* < 0.05 and FDR < 0.25 were considered statistically significant.

### 2.5 Analysis of the protein interaction network

The STRING website (https://string‐db.org) is a database to predict protein–protein interactions (PPIs) (including at least 6k proteins). The PPI network information map was obtained by entering the VEGFA gene into the search bar. A combined score >0.7 was considered a close relationship.

### 2.6 Analysis of tumour‐related immune infiltration

The Tumour Immune Estimation Resource Web Server (TIMER) is a comprehensive resource for systematic analysis of immune infiltration in different cancer types. To analyse the correlation between the expression of VEGFA and immune infiltration in HCC tissues, the first six types of immune cells, including B cells, CD4+ T cells, CD8+ T cells, neutrophils, macrophages and dendritic cells, were obtained from the TIMER database. Next, CD8+ T cell, CD4+ T cell and T regulatory cell infiltration and VEGFA expression were calculated through this website. Then, the relationship between each immune cell marker and VEGFA expression was analysed. Additionally, the coefficient value (*R*) and corresponding *p* value of the correlation between VEGFA and immune cell markers were obtained from GEPIA.

### 2.7 Cell culture and transfection

The human normal liver cell line (L02) and HCC cell lines (HepG2, HepG3B, Huh7, SNU449 and PLC) were cultured in DMEM supplemented with 10% foetal bovine serum (FBS, Corning) at 37°C and 5% CO_2_. Short interfering (si)RNA targeting vascular endothelial growth Factor A (si‐VEGFA) and siRNA negative control (si‐NC) were purchased from Guangzhou Sagene Biotech Co. Si‐VEGFA was transfected using lentivirus followed by subsequent experiments 48–72 h later.

### 2.8 RNA isolation, reverse transcription and qRT–PCR analysis

Total RNA was isolated from samples using TRIzol reagent (Invitrogen), and cDNA was obtained by using TransScript First‐Strand cDNA Synthesis SuperMix (TransGen). TransStart Green Q‐PCR SuperMix (TransGen) was used to perform quantitative real‐time PCR (qRT–PCR) according to the manufacturer's protocol as described in a previous study.^29^ The primers for the real‐time PCR were designed by Sangon Biotech. The primers used were 5′‐GCGGATCAAACCTCACCAAG‐3′ and 5′‐GCTTTCGTTTTTGCCCCTTTC‐3′ for VEGFA and 5′‐AATCCCATCACCATCTTCCA‐3′ and 5′‐CCTGCTTCACCACCTTCTTG‐3′ for GAPDH. Relative mRNA expression levels were normalized to GAPDH levels.

### 2.9 Protein extraction and immunoblottingtechniques

Protein extraction and immunoblotting were performed as described in a previous study.^30^ Briefly, RIPA lysis buffer was added to tissue or cell samples to obtain total protein. Then, 5× SDS buffer was added to quantify the protein samples at 99°C for 10 min to desaturate the protein. The proteins were separated by SDS–PAGE electrophoresis, transferred to PVDF membranes, blocked with 3% nonfat dry milk (PBST) for 1 h, incubated with a VEGFA antibody (AF5131, Affinity Biosciences) overnight at 4°C, washed three times with PBST, incubated with a secondary antibody for 90 min at room temperature and washed three times with PBST. Finally, chemiluminescence imaging was performed to detect protein expression levels on the membranes.

### 2.10 Immunohistochemistry

Immunohistochemistry (IHC) was performed as described in a previous study.^31^ Briefly, the tissue blocks were fixed with 4% polychloroformaldehyde, rinsed, dehydrated with gradient ethanol, embedded in paraffin and prepared into paraffin sections with a thickness of 4 μm for staining. Then, the paraffin sections were dewaxed with xylene, dehydrated with gradient ethanol, incubated with antibodies (primary antibody, secondary antibody), dehydrated, cleared, mounted and observed under a microscope.

### 2.11 Immunofluorescence technique

Paraffin sections were deparaffinized for antigen retrieval, blocked with hydrogen peroxide, and serum blocked. CD86 primary antibody (DF6332, Affinity Biosciences) was added overnight followed by secondary antibody incubation. After FITC‐TSA treatment and microwave treatment, CTLA4 primary antibody (DF6793, Affinity Biosciences) was added overnight followed by secondary antibody incubation. The nuclei were stained with DAPI, and the glass was mounted. Finally, the images were observed and collected under a fluorescence microscope.

### 2.12 Detection of apoptotic rate by flowcytometry

All the supernatant and adherent cells were collected. Annexin V/FITC staining was performed according to the instructions of the Annexin V/FITC Apoptosis Kit.

### 2.13 Transwell chamber experiment to detect the number of migrating cells

The cells were trypsinized, and 2.5 × 10^4^ cells were seeded into the upper chamber of the Transwell. Meanwhile, 500 μl of complete medium containing 10% FBS was added to the lower chamber. The cells were cultured for another 48 h. After that, the chamber was removed and washed with PBS. Next, the cells were fixed with 4% paraformaldehyde for 15 min, washed three times with PBS, stained with 0.1% crystal violet for 10 min and washed three times with PBS. Then, the pictures were selected randomly under a microscope. The number of migrated cells was counted. The experiments were repeated in triplicate.

### 2.14 Statistical analysis

Statistical analysis was performed using GraphPad Prism version 8.0 (GraphPad software) and SPSS 20.0 (SPSS). The measurement data are usually expressed as the mean ± SD. An independent samples *t*‐test was applied for the difference between the two groups. Welch's analysis of variance (Welch's anova) was used to analyse the difference between multiple groups. Spearman's rank correlation analysis was performed to assess the correlation of gene expression in tissue arrays. Other data were compared by Student's *t* test or the Mann–Whitney test. Two‐sided *p* values less than 0.05 were considered statistically significant.

## 3 RESULTS

### 3.1 The expression of VEGFA is higher in tumour tissues than in the corresponding normal tissues

**FIGURE 1:**
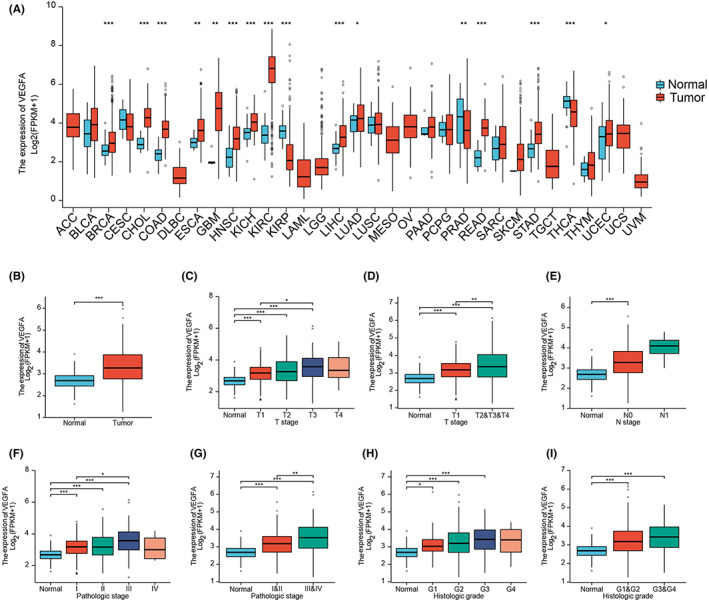
Expression of VEGFA in tumour and normal tissues. (A) VEGFA expression levels in different types of tumour and normal tissues from TCGA database. (B) VEGFA expression levels in HCC from TCGA database. (C–I) VEGFA expression in different stages of HCC from TCGA database. *p < 0.05, **p < 0.01, ***p < 0.001. ACC, adrenocortical carcinoma; BLCA, bladder urothelial carcinoma; BRCA, breast invasive carcinoma; CHOL, cervical and endocervical cancers (CESC), cholangiocarcinoma; COAD, colon adenocarcinoma; DLBC, lymphoid neoplasm diffuse large B‐cell lymphoma; ESCA, oesophageal carcinoma; GBM, glioblastoma multiforme; HNSC, head and neck squamous cell carcinoma; KICH, kidney chromophobe; KIRC, kidney renal clear cell carcinoma; KIRP, kidney renal papillary cell carcinoma; LAML, acute myeloid leukaemia; LGG, brain lower grade glioma; HCC, hepatocellular carcinoma; LUAD, lung adenocarcinoma; LUSC, lung squamous cell carcinoma; MESO, mesothelioma; OV, ovarian serous cystadenocarcinoma; PAAD, pancreatic adenocarcinoma; PCPG, pheochromocytoma and paraganglioma; PRAD, prostate adenocarcinoma; READ, rectum adenocarcinoma; SKCM, skin cutaneous melanoma; STAD, stomach adenocarcinoma; TGCT, testicular germ cell tumours; THCA, thyroid carcinoma; UCEC, uterine corpus corpus endometrial carcinoma

We analysed the expression of VEGFA mRNA in different human tumour tissues and corresponding normal tissues from the TCGA database and GEO database. Additionally, we analysed the expression of VEGFA protein in liver cancer tissue and adjacent tissue. From the TCGA database, we collected a total of 424 samples, including 374 HCC specimens and 50 normal tissue specimens. All the sample information was also collected, including RNA‐sequencing data and detailed clinical prognostic information resources (Table 1). The results from TCGA data showed that the expression of VEGFA mRNA in most tumour tissues was significantly higher than that in corresponding normal tissues, including BRCA, CHOL, COAD, ESCA, GBM, HNSC, KICH, KIRC, LIHC, LUAD, PRAD, READ, STAD and UCEC (*p* < 0.05; Figure 1A,B). Therefore, we further analysed the correlation between patients with HCC with different clinicopathological features and VEGFA mRNA expression in which VEGFA gene expression level had no significant correlation with M stage but was correlated with T stage, N stage, pathologic stage and histologic grade (Figure 1C–I). Especially for the relationship of pathologic grade and VEGFA expression (*p* < 0.01), the higher the pathological stage was, the higher the expression level of VEGFA. All these data indicated that VEGFA expression was relatively higher in patients with HCC with poor prognosis.

**TABLE 1:** Clinical characteristics of the patients with HCC

Characteristic	Low expression of VEGFA	High expression of VEGFA	*p*
*n*	187	187	
Gender, *n* (%)			
Female	43 (11.5%)	78 (20.9%)	<0.001
Male	144 (38.5%)	109 (29.1%)	
Age (years), *n* (%)			
<=60	96 (25.7%)	81 (21.7%)	0.133
>60	90 (24.1%)	106 (28.4%)	
Height (cm), *n* (%)			
<170	91 (26.7%)	110 (32.3%)	0.010
> = 170	84 (24.6%)	56 (16.4%)	
Weight (kg), *n* (%)			
<=70	84 (24.3%)	100 (28.9%)	0.050
>70	92 (26.6%)	70 (20.2%)	
BMI (kg/cm^2^), *n* (%)			
<=25	92 (27.3%)	85 (25.2%)	0.981
>25	82 (24.3%)	78 (23.1%)	
AFP (ng/ml), *n* (%)			
<=400	114 (40.7%)	101 (36.1%)	0.139
>400	27 (9.6%)	38 (13.6%)	
Child‐Pugh grade, *n* (%)			
A	118 (49%)	101 (41.9%)	0.647
B	12 (5%)	9 (3.7%)	
C	0 (0%)	1 (0.4%)	
T stage, *n* (%)			
T1	103 (27.8%)	80 (21.6%)	0.035
T2	47 (12.7%)	48 (12.9%)	
T3	30 (8.1%)	50 (13.5%)	
T4	5 (1.3%)	8 (2.2%)	
N stage, *n* (%)			
N0	124 (48.1%)	130 (50.4%)	0.623
N1	1 (0.4%)	3 (1.2%)	
M stage, *n* (%)			
M0	134 (49.3%)	134 (49.3%)	1.000
M1	2 (0.7%)	2 (0.7%)	
Pathologic stage, *n* (%)			
Stage I	96 (27.4%)	77 (22%)	0.048
Stage II	45 (12.9%)	42 (12%)	
Stage III	32 (9.1%)	53 (15.1%)	
Stage IV	3 (0.9%)	2 (0.6%)	
Histologic grade, *n* (%)			
G1	33 (8.9%)	22 (6%)	0.115
G2	95 (25.7%)	83 (22.5%)	
G3	53 (14.4%)	71 (19.2%)	
G4	5 (1.4%)	7 (1.9%)	
Residual tumour, *n* (%)			
R0	166 (48.1%)	161 (46.7%)	0.044
R1	4 (1.2%)	13 (3.8%)	
R2	1 (0.3%)	0 (0%)	
Vascular invasion, *n* (%)			
No	106 (33.3%)	102 (32.1%)	1.000
Yes	56 (17.6%)	54 (17%)	

**FIGURE 2:**
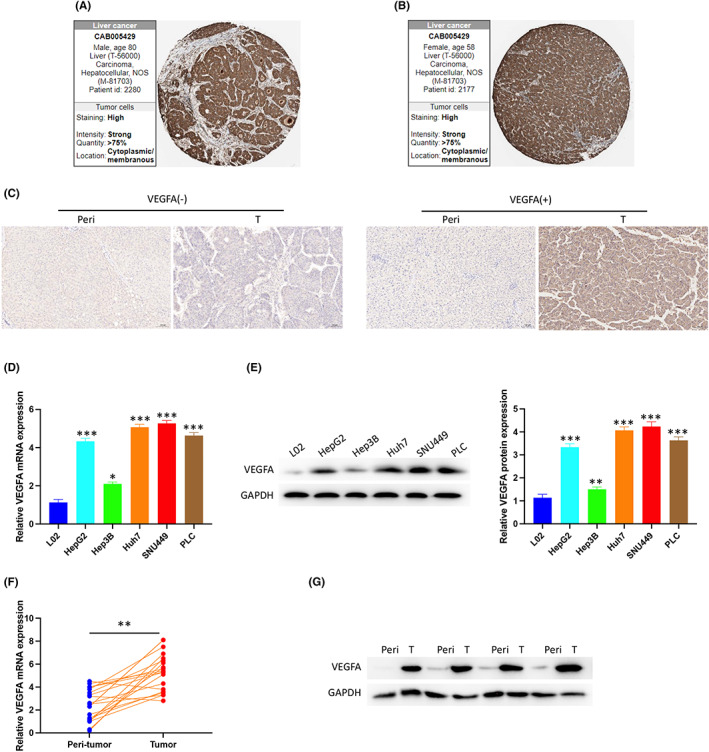
Expression of VEGFA in HCC. (A, B) VEGFA was highly expressed in HCC tumours from the HPA. (C) VEGFA expression in peritumoral and HCC tissues by immunohistochemistry (IHC). (D) VEGFA mRNA expression in normal and HCC cells was detected by PCR. (E) VEGFA protein expression in normal hepatocytes and HCC cells was detected by Western blotting. (F) VEGFA mRNA expression in peritumoral and HCC tissues was detected by PCR. (G) VEGFA protein expression in peritumoral and HCC tissues was detected by Western blot. peri, peritumoral tissue; T, tumour tissue

We further collected the IHC profile of VEGFA protein in the HPA database. The results suggested that VEGFA protein expression was high in the majority of HCC tissues (Figure 2A,B). To further confirm the reliability of the public database, we first collected tumours and peritumoral tissues from 60 patients with HCC in the Department of Hepatobiliary Surgery of Hunan Provincial People's Hospital for IHC staining. Among them, 51 tumour tissues and three peritumoral tissues showed high expression of VEGFA. No or low expression of VEGFA was found in nine tumour tissues and 57 peritumoral tissues. We present the results of VEGFA‐negative and VEGFA‐positive expression in tumour tissue and peritumoral tissue, respectively. (Figure 2C). The expression of VEGFA mRNA and protein in tumour tissues and HCC cells (HepG2, HepG3B, Huh7, SNU‐449 and PLC) was significantly higher than that in adjacent tissues and normal liver cells (L02) by PCR and Western blotting (Figure 2D–G).

### 3.2 VEGFA has good diagnostic sensitivity in HCC

**FIGURE 3:**
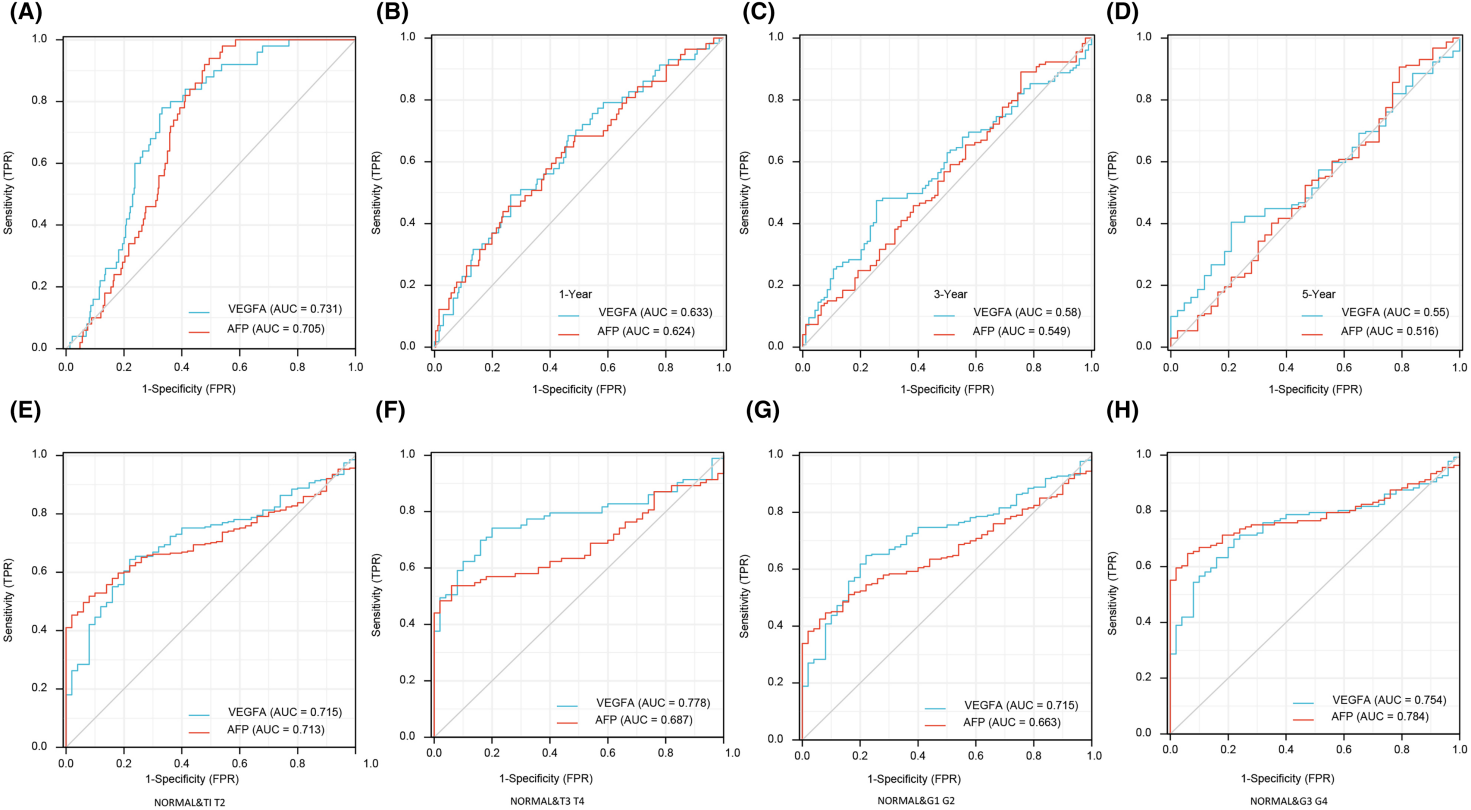
ROC curve was established by TCGA Program database. (A) Diagnostic efficacy of VEGFA and AFP in HCC. (B–D) Diagnostic efficacy of VEGFA and AFP in different stages of HCC. (E–H) Differences in the diagnosis of VEGFA and AFP between normal patients and patients with HCC at different timepoints

In this study, we assessed the diagnostic value of VEGFA in HCC by generating ROC curves from the TCGA database. The results showed that the area under the curve (AUG) of VEGFA was 0.731 (Figure 3A), and to some extent, the diagnostic performance of VEGFA was no less than that of AFP. In addition, we also analysed the diagnostic value of VEGFA in different stages of HCC. The results showed AUG = 0.633 at 1 year of tumour progression (Figure 3B), AUG = 0.58 at 3 years of tumour progression (Figure 3C), AUG = 0.55 at 5 years of tumour progression (Figure 3D), AUG = 0.715 at the T1 and T2 stages (Figure 3E), AUG = 0.778 at the T3 and T4 stages (Figure 3F), AUG = 0.715 at the G1 and G2 stages (Figure 3G), and AUG = 0.754 at the G3 and G4 stages (Figure 3H). All of these data support that VEGFA might be a potential new biomarker.

### 3.3 Higher expression levels of VEGFA mRNA are associated with worse prognosis

**FIGURE 4:**
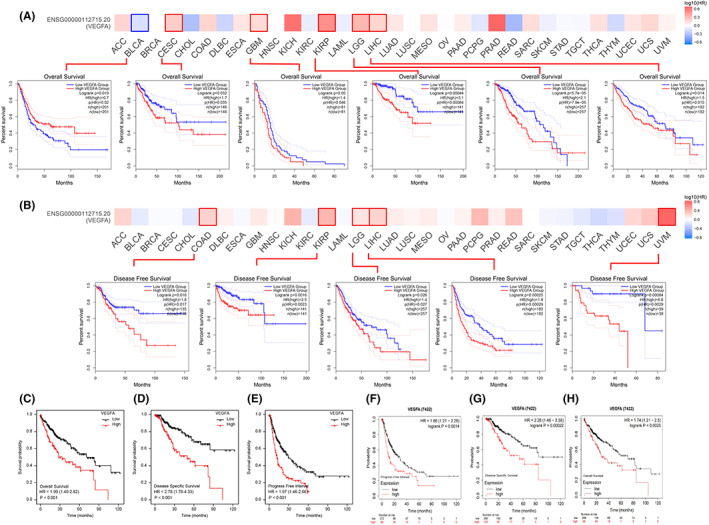
Kaplan–Meier survival curve analysis of the prognostic significance of VEGFA expression in different types of human cancers. (A) The correlation between VEGFA expression levels and OS in different tumours was analysed using the GEPIA2 website, TGCA database and GTEx database. (B) The correlation between VEGFA expression levels and DFS in different tumours was analysed using the GEPIA2 website, TGCA database and GTEx database. (C–E) The expression level of VEGFA was negatively correlated with OS, DSS and PFI of HCC by TCGA database. (F–H) Kaplan–Meier Plotter was used to analyse the expression levels of VEGFA in the GEO, EGA and TCGA databases, and there was a negative correlation with OS, DSS and PFI of HCC

The GEPIA2 website was applied to comprehensively analyse the information of the TCGA database and GTEx database. The results showed that a high VEGFA mRNA expression level was associated with shorter overall survival of CESC, GBM, KIRP and LIHC (*p* < 0.05; Figure 4A). Moreover, a high expression level of VEGFA mRNA was associated with shorter disease‐free survival in COAD, KIRP, LGG, LIHC and UVM (*p* < 0.05; Figure 4B). Unexpectedly, low VEGFA mRNA expression was associated with shorter overall survival in BLCA (*p* < 0.05; Figure 4A). All the above results indicated that the expression of VEGFA in different tumour patients had different prognoses. A high expression level of VEGFA mRNA in tumour tissues indicates a poor prognosis. Next, TCGA database was applied to verify the correlation between the expression level of VEGFA mRNA and the prognosis of patients with HCC. The results showed that the overall survival, disease‐free survival rate and progression‐free interval of patients with HCC with high VEGFA mRNA expression were lower than those with low VEGFA mRNA expression (Figure 4C–E). Univariate and multivariate Cox regression analyses proved that the expression level of VEGFA may be negatively correlated with the prognosis of patients (Table 2). Finally, the above results were verified again by using the Kaplan–Meier Plotter website to synthesize the GEO, EGA and TCGA databases (Figure 4F–H).

**TABLE 2:** Correlations between overall survival and mRNA expression of VEGFA analysed by univariate and multivariate Cox regression

Characteristics	Total (*N*)	Univariate analysis		Multivariate analysis	
		Hazard ratio (95% CI)	*p* value	Hazard ratio (95% CI)	*p* value
Age	373				
<=60	177	Reference	0.295		
>60	196	1.205 (0.850–1.708)			
T stage	370				
T1&T2	277	Reference	＜0.001		0.645
T3&T4	93	2.598 (1.826–3.697)		1.603 (0.215–11.974)	
N stage	258				
N0	254	Reference	0.324		
N1	4	2.029 (0.497–8.281)			
M stage	272				
M0	268	Reference	0.017		0.226
M1	4	4.077 (1.281–12.973)		2.089 (0.634–6.889)	
Pathologic stage	349				
Stage I & Stage II	259	Reference	<0.001		0.592
Stage III & Stage IV	90	2.504 (1.727–3.631)		1.736 (0.230–13.082)	
Histologic grade	368				
G1&G2	233	Reference	0.636		
G3&G4	135	1.091 (0.761–1.564)			
Adjacent hepatic tissue inflammation	236				
None	118	Reference	0.475		
Mild&Severe	118	1.194 (0.734–1.942)			
Vascular invasion	317				
No	208	Reference	0.163		
Yes	109	1.344 (0.887–2.035)			
Gender	373				
Female	121	Reference	0.200		
Male	252	0.793 (0.557–1.130)			
VEGFA	373	1.331 (1.071–1.655)	0.010	1.028 (0.780–1.354)	0.845

### 3.4 Network enrichment analysis identifies VEGFA functions, associated signalling pathways and genes

**FIGURE 5:**
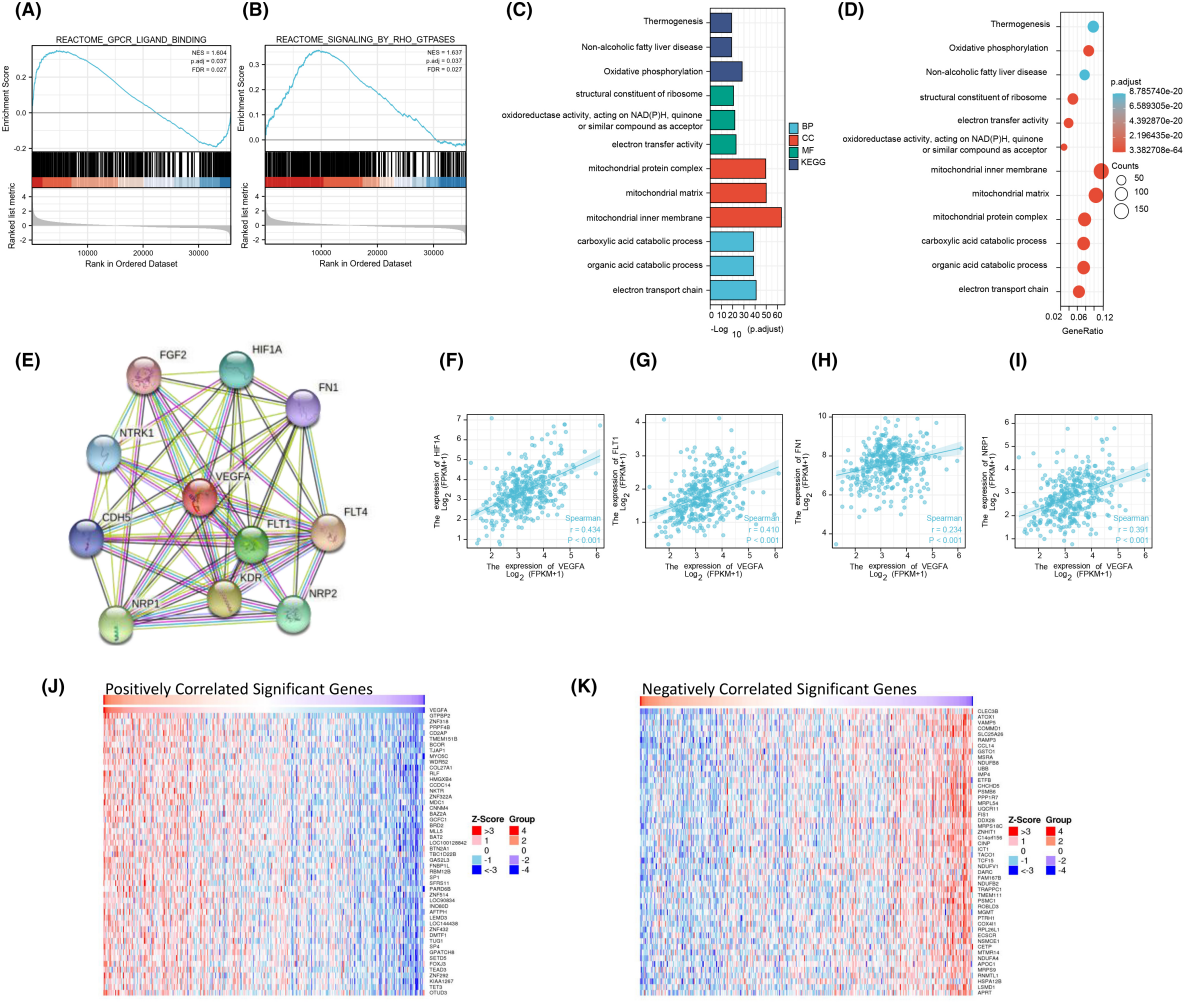
Enrichment analysis of VEGFA functional networks. (A, B) Enrichment plots by GSEA. (C, D) Enrichment of GO terms and KEGG for genes related to VEGFA. (E) PPI network of VEGFA. (F–I) Correlation between VEGFA expression levels and HIF1A, FLT1, FN1 and NRP1 expression levels. (J) The heatmap shows the top 50 genes positively related to VEGFA in the HCC cohort. (K) The heatmap shows the top 50 genes negatively related to VEGFA in the HCC cohort

Through GSEA, it was shown that VEGFA was involved in the two pathways of GPCR ligand binding and rho GTPases (Figure 5A,B). The results of GO KEGG analysis showed the signalling pathway, cellular component (CC), biological process and molecular function of VEGFA enrichment. GO analysis suggested that VEGFA was involved in carboxylic acid catabolic processes, organic acid catabolic processes, electron transport chains and other signalling pathways. Additionally, VEGFA participated in structural constituents of ribosomes; oxidoreductase activity; acting on NAD(P)H, quinone, or similar compounds as acceptors; and electron transfer activity. Moreover, it plays an important role in the formation of the mitochondrial protein complex, mitochondrial matrix, mitochondrial inner membrane and other CCs. Furthermore, KEGG analysis indicated that VEGFA was involved in biological activities such as thermogenesis, nonalcoholic fatty liver disease and oxidative phosphorylation (Figure 5C,D).

To study the interaction between VEGFA and other molecules in HCC, we obtained an interaction network map between VEGFA‐related proteins from the STRING website to further study the role of VEGFA in HCC. Among them, the following ten proteins, HIF1A, FN1, FLT4, NRP2, FLT1, KDR, NRP1, CDH5, NTRK1 and FGF2, had intensive interaction with VEGFA proteins (Figure 5E). The expression levels of HIF1A, FLT1, FN1 and FGF2 were positively correlated with the expression of VEGFA (Figure 5F–I). In addition, the top 50 genes positively and negatively correlated with VEGFA gene expression are also shown in a heatmap (Figure 5J,K). All the results provide new information for an in‐depth understanding of VEGFA.

### 3.5 VEGFA is related to tumour immune cell infiltration and the immune microenvironment

**FIGURE 6:**
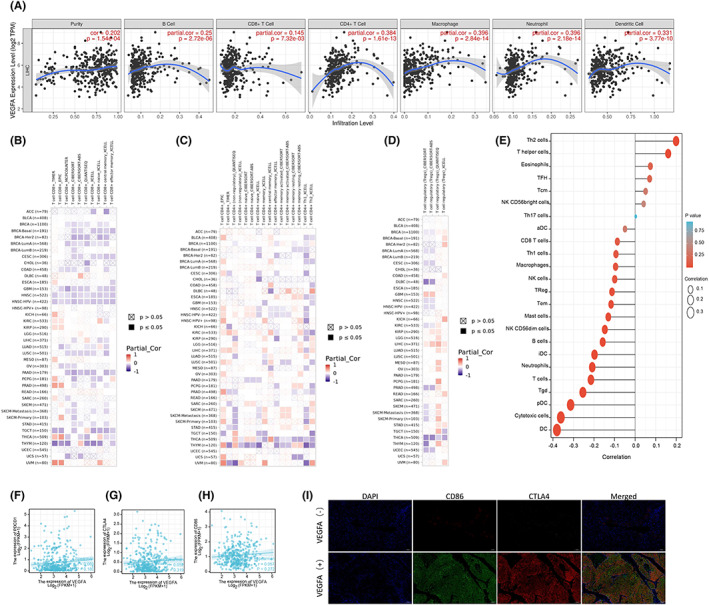
Correlation analysis of VEGFA expression and infiltration levels of immune cells in tumour tissues. (A) VEGFA expression was positively correlated with tumour purity and infiltration levels of B cells, CD8^+^ T cells, CD4^+^ T cells, macrophages and DCs in HCC tissues based on the TIMER database. (B–D) The relationship between VEGFA mRNA expression and CD8^+^ T cells, CD4^+^ T cells and regulatory T cells in various tumours was evaluated using a variety of algorithms based on the TIMER database. (E) Correlation between VEGFA expression level and infiltration of various immune cells (Th2 Cell, T helper Cell, Eosinophils, TFH, Tcm, NK CD58bright Cell, Th17‐cell, sDC, CD8^+^ T‐cell, Th1‐Cell, Macrophages, NK‐Cell, Treg, Tem, Mast cell, NK CD58dim Cell, B‐cell, iDC, Neutrophils, T‐cell, Tgd, pDC, Cytotoxic Cell, DC) in HCC tissues. (F–H) Correlation between VEGFA expression level and PDCD1, CTLA4 and CD86 expression levels in HCC tissues based on TCGA database. (I) The correlation between VEGFA expression levels and PDCD1, CTLA4 and CD86 expression levels in HCC tissues was detected by immunofluorescence

It is well known that tumour‐infiltrating lymphocytes influence the development of human tumours and affect the survival time of cancer patients. We first used the TIMER database to analyse the correlation of VEGFA expression with tumour purity and infiltration of six types of immune cells: CD8^+^ T cells, CD4^+^ T cells, B cells, dendritic cells, macrophages and neutrophils. The results showed that the high expression of VEGFA had a significant positive correlation with the high infiltration of the above six types of immune cells among which there was a correlation with B cells (cor = 0.25, *p* = 2.72e−06), CD8^+^ T cells (cor = 0.145, *p* = 7.32e−03), CD4^+^ T cells (cor = 0.384, *p* = 2.84e−14), macrophages (cor = 0.396, *p* = 2.18e−14), neutrophils (cor = 0.396, *p* = 2.18e−14) and dendritic cells (cor = 0.331, *p* = 3.77e−10; Figure 6A). In addition, we used other algorithms to evaluate the relationship between VEGFA expression and the infiltration of immune cells, including CD8^+^ T cells, CD4^+^ T cells and regulatory T cells, in various tumours (Figure 6B–D). Furthermore, various immune cells (Th2 cells, T helper cells, eosinophils, TFH cells, Tcm cells, NK CD58bright cells, Th17 cells, sDCs, CD8 T cells, Th1 cells, macrophages, NK cells, Tregs, Tems, mast cells, NK infiltration of CD58dim cells, B cells, iDCs, neutrophils, T cells, Tgd cells, pDCs, cytotoxic cells and DCs) in HCC tissues were also analysed (Figure 6E). To further understand the correlation between VEGFA and immunotherapy, we analysed the relationship of VEGFA and PDCD1, CD86, and CTLA4, which are important markers of the immunosuppressive microenvironment, from the TCGA database. The results showed that VEGFA was positively correlated with these proteins (Figure 6F–H). Furthermore, we verified this finding by immunofluorescence of HCC tissues. This result was consistent with the TCGA database in which VEGFA‐positive tumours had stronger expression of CD86 and CTLA4 suggesting that VEGFA was related to the immunosuppressive microenvironment (Figure 6I).

### 3.6 Inhibition of VEGFA reduces proliferation, invasion and migration and promotes apoptosis in HCC cells

**FIGURE 7:**
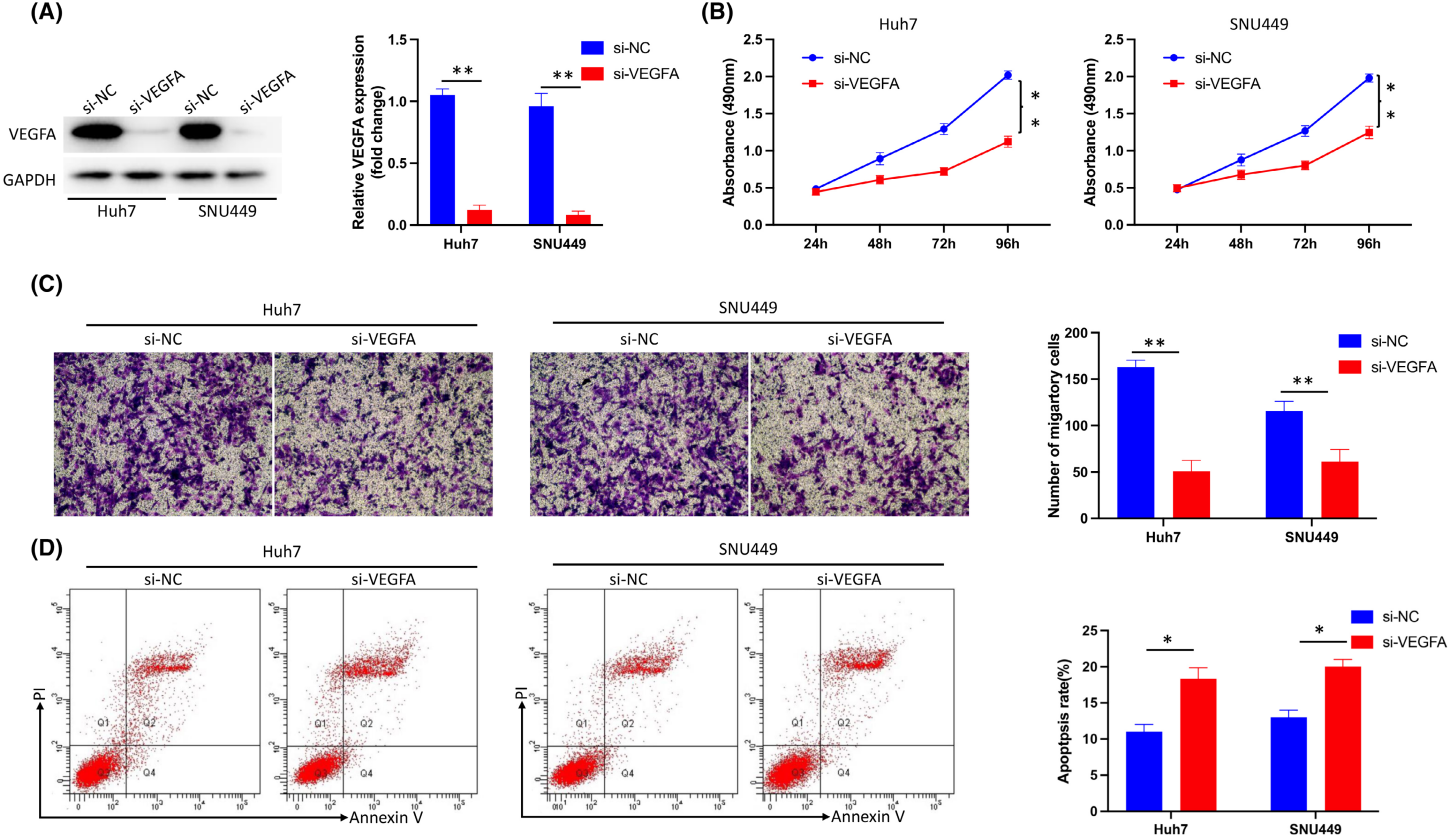
Inhibition of VEGFA reduced the proliferation, invasion and migration of HCC cells and promoted apoptosis. (A) Transfection efficiency of si‐VEGFA in Huh7 and SUN449 cells. (B) Inhibition of VEGFA significantly inhibited the proliferation of HCC cells as shown by CCK8 assay. (C) Inhibition of VEGFA significantly inhibited HCC migration as shown by transwell assays. (D) Inhibition of VEGFA promoted apoptosis of HCC cells

To explore the effect of VEGFA in HCC, we transfected si‐VEGFA into HCC cells (Figure 7A) and detected the proliferation ability of the cells by CCK8. The results showed that inhibition of VEGFA could significantly inhibit the proliferation of HCC (Figure 7B). Next, the cell migration ability was assessed by transwell assay, and the results suggested that the inhibition of VEGFA could significantly inhibit the migration ability of HCC (Figure 7C). Furthermore, cell apoptosis was calculated by FCM, and the results showed that inhibiting VEGFA could induce increased apoptosis. (Figure 7D).

## 4 DISCUSSION

VEGFA, also known as vascular permeability factor, is considered to be a regulator of renal growth factor and vascular permeability.^32^ Cumulative evidence has shown that VEGF plays an important role in cancer progression.^33^, ^34^ In this study, we found that VEGFA was overexpressed in HCC tissues compared to normal tissues. Moreover, our results indicate that VEGFA is a potential prognostic biomarker and correlates with immune cell infiltration in HCC. This finding provides new insight into the combination of immunotherapy for HCC.

It is well known that AFP is an important tumour marker for the diagnosis of HCC.^35^, ^36^ In this study, we also found that VEGFA had a diagnostic specificity for HCC similar to that of AFP. This result indicated that VEGFA might be a potential new biomarker for the diagnosis and prognosis of HCC. Moreover, VEGFA was significantly higher in tumour tissue than in normal tissue suggesting that it played an important role in HCC. The higher expression of VEGFA in tumours with higher T stage and pathologic stage suggested that angiogenesis is an important factor in tumour growth and progression. However, there was no significant difference in the expression of VEGFA between the histologic grades indicating that VEGFA might not be related to tumour cell differentiation. Considering that higher expression of VEGFA is mostly associated with more advanced‐stage tumours and that advanced‐stage tumours often lose the opportunity for surgery, VEGFA is a good therapeutic target for these patients. Sorafenib is a multitarget antitumor drug that can also inhibit the VEGFR signalling pathway and angiogenesis.^37^ The SHARP Investigators Study Group found that sorafenib monotherapy for advanced HCC was significantly better than placebo.^38^ To date, targeted therapy drugs represented by sorafenib have dominated drug treatment in advanced HCC for many years. In the past 5 years, a variety of internationally recognized targeted drugs have emerged, such as lenvatinib, cabozantinib, regorafenib and ramucirumab.^39^, ^40^, ^41^, ^42^ Although the diversity of medicines has increased, other treatment options are lacking when targeted therapy fails.

Since 2017, immunotherapy has become another major breakthrough in advanced HCC.^43^ Although this method has shown good results in treatment, it is ineffective when applied alone.^44^, ^45^ Many studies on the prediction of immunotherapy have focused on biomarkers to guide clinical treatment.^28^, ^46^ Our results showed that the expression level of VEGFA was correlated with immune cell infiltration, including M2 TAMs and Tregs, which were associated with promoting cancer progression.^47^, ^48^ Moreover, the expression level of VEGFA mRNA was also positively correlated with CD86 and CTLA4. Furthermore, we verified this finding by immunofluorescence. It has also been reported that VEGF can reduce the ability of antigen‐presenting cells to activate T cells, increase Treg cells and promote the polarization of TAMs to the M2 phenotype suggesting that inhibition of the VEGFR signalling pathway can reshape the immune microenvironment,^49^, ^50^, ^51^ and combined immunotherapy can achieve a synergistic result. In fact, there have been relevant studies on combined therapy, and preliminary results have shown promising results.^52^, ^53^

Previous studies have shown that VEGFA ultimately leads to cell proliferation, cell survival, cell migration, vascular permeability, invasion of surrounding tissues and endothelial inflammation thereby achieving angiogenesis through a series of VEGFA‐induced signalling pathways, such as the phospholipase Cγ (PLCγ)–extracellular regulated kinase pathway, src kinases, focal adhesion kinase, the PI3K‐Akt pathway and the Rho family of monomeric G proteins (GTPases).^54^, ^55^, ^56^ In this study, we also found that VEGFR played an important role in GPCR ligand binding and rho GTPases. A PPI study showed that VEGFA might have a close interaction with HIF1A, FLT1, FN1 and FGF2. All these molecules can promote HCC development by activating various pro‐tumour signals.^57^, ^58^, ^59^, ^60^ We also verified the function of VEGFA in HCC cell lines by silencing its expression. Silencing the expression of VEGFA can inhibit the proliferation of HCC cells, inhibit the invasive ability and promote cell apoptosis. This is a potential antitumour effect in addition to the effect of VEGFA binding to VEGFR on endothelial cells to promote angiogenesis. Therefore, VEGFA is an important factor that promotes HCC and may be used as a diagnostic indicator that is similar to AFP in the future. Additionally, it can predict the prognosis of HCC, and its high expression has a worse prognosis. Furthermore, it is also related to immune infiltration suggesting that targeted inhibition of VEGFA and combined immune treatment is a viable strategy.

## 5 CONCLUSION

In this study, we found that VEGFA is a potential predictive biomarker for the diagnosis and prognosis of HCC through the TCGA database and further molecular biology experiments. Although this finding is valuable, the specific mechanism by which VEGFA affects HCC immunotherapy was not explored in depth in this study. In future studies, we will focus on clarifying the possibility of VEGFA as a biomarker for HCC immunotherapy.

## AUTHOR CONTRIBUTIONS

**Yuchen Qi:** Conceptualization (equal); data curation (equal); formal analysis (equal); methodology (equal); visualization (equal); writing – original draft (equal). **Yinghui Song:** Conceptualization (equal); data curation (equal); formal analysis (equal); methodology (equal); resources (equal); visualization (equal); writing – original draft (equal). **Mengting Cai:** Formal analysis (equal); methodology (equal); visualization (equal); writing – original draft (equal). **Jianwen Li:** Supervision (equal); writing – review and editing (equal). **Zhangtao Yu:** Data curation (equal); investigation (equal). **Yuhang Li:** Methodology (equal); writing – original draft (equal). **Junkai Huang:** Methodology (equal); writing – original draft (equal). **Chuang Peng:** Writing – review and editing (equal). **Bo Jiang:** Writing – review and editing (equal). **Sulai Liu:** Conceptualization (lead); project administration (equal); writing – review and editing (equal). **Yu Jiang:** Data curation (equal); writing – original draft (equal).

## CONFLICT OF INTEREST STATEMENT

The authors confirm that there are no conflicts of interest.

## INFORMED CONSENT

Written informed consent for publication was obtained from all participants.

## Data Availability

The data that support the findings of this study are available from the corresponding author upon reasonable request.
